# HyperISGylation of Old World Monkey ISG15 in Human Cells

**DOI:** 10.1371/journal.pone.0002427

**Published:** 2008-06-18

**Authors:** Els Pattyn, Annick Verhee, Isabel Uyttendaele, Julie Piessevaux, Evy Timmerman, Kris Gevaert, Joël Vandekerckhove, Frank Peelman, Jan Tavernier

**Affiliations:** 1 Department of Medical Protein Research, VIB, Ghent, Belgium; 2 Department of Biochemistry, Ghent University, Ghent, Belgium; Karolinska Institutet, Sweden

## Abstract

**Background:**

ISG15 is an Ubiquitin-like protein, highly induced by Type I Interferons. Upon the cooperative activity of specific Ubiquitinating enzymes, ISG15 can be conjugated to its substrates. Increasing evidence points to a role for protein ISGylation in anti-viral and anti-tumoral defense.

**Principal Findings:**

We identified ISG15 from Old World Monkeys (OWm) as a hyper-efficient protein modifier. Western blot analysis visualized more efficient conjugation of OWmISG15 relative to HuISG15 in human (Hu), monkey and mouse (Mo) cell-lines. Moreover, the substrates of OWmISG15 identified upon Tandem Affinity Purification followed by LC-MS/MS identification largely outnumbered these of HuISG15 itself. Several Ubiquitin-Conjugating enzymes were identified as novel ISGylated substrates. Introduction of a N89D mutation in HuISG15 improved its ISGylation capacity, and additional Q31K/T33A/D133N mutations yielded a HuISG15 variant with an ISGylation efficiency comparable to OWmISG15. Homology modeling and structural superposition situate N89 in the interaction interface with the Activating enzyme. Analysis of the UbE1L residues in this interface revealed a striking homology between OWmUbE1L and HuUbE1, the Activating enzyme of Ubiquitin. In line with this observation, we found efficient activation of AgmISG15, but not HuISG15 or MoISG15, by HuUbE1, thus providing a likely explanation for OWm hyperISGylation.

**Conclusions:**

This study discloses the poor conjugation competence of HuISG15 compared to OWmISG15 and maps the critical determinants for efficient conjugation. HyperISGylation may greatly assist ISGylation studies and may enhance its function as positive regulator of Interferon-related immune responses or as anti-tumoral modulator.

## Introduction

Type I Interferons (IFN)s are involved in host defense mechanisms, particularly against viral infections. They induce a so-called “antiviral state” by inducing both cytosolic and nuclear events. IFN Stimulated Gene 15 (ISG15) is an Ubiquitin (Ub)-Like molecule (UbL), highly induced upon both type I and type II IFN treatment [1]. It is expressed as a 17 kDa protein, containing 2 Ub domains and a C-terminal oligopeptide (eg. octapeptide in human, hexapeptide in mice). Maturation of ISG15 includes N-terminal Met excision [2] and removal of the C-terminal peptide giving a 15 kDa protein [3]. Maturated ISG15 can then be conjugated via an isopeptide binding to the ε-amino group of a Lys residue in the target protein [4]. Alternatively, the processed 15 kDa ISG15 molecule can be secreted and exerts immunoregulatory functions on peripheral blood lymphocytes [5].

Conjugation of Ub(L) requires the cooperative activity of at least 3 enzymes. The ubiquitination cascade is initiated by an Ub-Activating enzyme (termed Uba, Ube or E1) adenylating the C-terminus of Ub(L), thereby forming an acyl-phosphate linkage with AMP. The catalytic Cys residue in UbE1 subsequently attacks this high energy bond, forming a thiolester bond to the C-terminal Gly of Ub(L). In humans, Ub molecules are found to be activated by UbE1 (also known as A9S1) [6] or UbE1L2 (also named Uba6) [7], [8], ISG15 by UbE1L [9], SUMO by AOS-Uba1 [10] and Nedd8 by AppBp1-Uba3[11]. Ub(L) molecules thiolester-linked to its Ub-Activating enzyme are transferred to a Ub-Conjugating enzyme (termed Ubc or E2), also by a thiolester linkage on a Cys residue. UbcH8 has been identified as a major Ub-Conjugating enzyme effecting ISG15 conjugation [12], [13]. Around 400 proteins are recognized as Ub-Ligases or E3s. Roughly, they can be discerned as RING (Really Interesting New Gene)-finger proteins, acting as a molecular scaffold, and HECT (Homologous to E6-AP C-Terminus)-domain proteins, which also exert a catalytic contribution. Ub-Ligases confer specificity, and place the Ub or UbL molecule in close proximity to the Lys residue of the substrate. The formation of polyubiquitin chains is a process mediated by the Ub-Conjugating enzyme together with the Ub-Ligase. Recently, the IFN-induced HERC5 has been identified as an ISG15 E3-Ligase in human cells [14]. The Estrogen-response Finger Protein (EFP), also an IFN-induced protein, functions as an E3-Ligase for ISGylation of 14-3-3δ [15]. Definitely, these recently discovered ISG15-Ligases are only the onset of a more extensive list. As Ub, most UbLs are synthesized as inactive precursors, being processed by De-UBiquitinating enzymes (DUBs) exposing the mature protein with a C-terminal Gly residue. DUBs not only exert their function by protein processing, they also have a function in removal of the Ub(L) from their substrate. Ub Specific Protease 2 (USP2), USP5 (also named Isopeptidase T), USP13 (IsoT3) and USP14 have been identified as proteases with a dual specificity for Ub and ISG15 [16], [17]. USP18 (also named UBP43) specifically cleaves ISG15 [18]. Of note, USP18 also competes with Jak1 for binding to the Type I IFN receptor IFNaR2 [19].

ISG15 is upregulated upon viral infection. Its role in antiviral defense is underscored by viral mechanisms to counteract ISG15 function. For example, the influenza B Non-Structural NS1B protein binds ISG15, thereby preventing its association to UbE1L [9]. Also the papain-like protease of the Severe Acute Respiratory Syndrome (SARS) Coronavirus, counteracts ISG15 functioning by removing it from its substrate [20]. The Hepatitis C virus NS3/4A protease cleaves IFN Promoter-Stimulator-1 (IPS-1) leading to reduced ISG15 expression and conjugation [21]. In a more direct way, ectopic expression of ISG15 in cells lacking a functional IFN response reduces Newcastle Disease Virus and Influenza Virus replication [22], and also overcomes fatal intracerebral infection in IFNaR^−/−^ mice by Sindbis Virus [23]. ISG15 knock-out mice proved to be more sensitive to Influenza, Herpes and Sindbis viral infection [24]. An inverse dose-response correlation of exogenously expressed ISG15 and release of Human Immunodeficiency Virus (HIV) virions is also found and suppression of ISG15 expression by siRNA counteracts IFN-mediated inhibition of HIV virion release [25].

The precise mode of action of ISG15 is not yet established. ISGylation is believed to counteract Ubiquitination by competing for the same Lys in substrates [26]. Studies with UbcH6 and UbcH13 showed that ISGylation of these E2s hampered their ability to form a thiolester intermediate with Ub [27], [28]. This competition may protect substrate proteins from proteasomal degradation.

We here report the surprising finding that ISG15 from Old World monkeys is a far more efficient protein modifier than its human counterpart in human, monkey and mouse cells. We identified novel ISGylation substrates and mapped the critical residues in this ISGylation process. This hyperISGylation competence of AgmISG15 was validated by an alternative activation mechanism. These findings will be useful for a better understanding of ISG15 biology.

## Results

### ISG15 orthologues exhibit large sequence divergence

**Figure 1:**
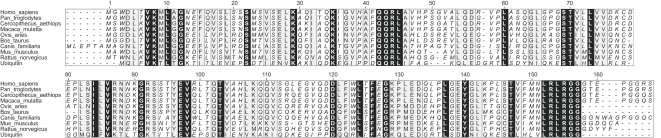
ISG15 orthologues show a low degree of amino acid conservation. ISG15 from different species were aligned with two molecules of Ubiquitin using Clustalx [61]. Sequence conservation is visualized using the AMAS server [62] with a conservation threshold of 7. Residues with a conservation above this threshold are shown on a grey background, residues that are identical in all sequences have a black background.

Viral resistance strongly varies between species. In this respect, Old World Monkeys (OWm)s receive much attention by their remarkable HIV resistance. They are distinguished from Apes (here represented by the Chimpanzee) in that most have tails and are distinguished from New World Monkeys (not included in this study) in that their tails are never prehensile. We compared ISG15 sequences from hominid origin with those of OWm, which diverged some 25 million years ago, and of murine origin, which diverged from primates approximately 75 million years ago [29]. ISG15 from different origins were aligned to two Ub molecules (Figure 1). The N- and C-terminal part of ISG15 share respectively 32 and 37% sequence identity with Ub. The ISG15 variability largely exceeds what can be expected by genetic drift alone [2].

We isolated ISG15 cDNAs of Humans (allelic variant with N at position 83 and S at position 94) (*Homo sapiens*, hereafter HuISG15), Chimpanzee (*Pan troglodytes*, CpzISG15), African Green Monkey (*Cercopithecus aethiops*, AgmISG15), Rhesus Monkey (*Macaca mulatta*, RhmISG15) and Mouse (*Mus musculus*, MoISG15). All were C-terminally cloned after a FLAG recognition tag. HuISG15 and CpzISG15 only differ by one amino acid (respectively a Ser and Asn residue at position 21) and represent hominid ISG15 (HOmISG15). AgmISG15 and RhmISG15 differ from each other by two amino acids (respectively a Ser and Asn residue at position 21 and Arg and Lys at position 77), and are both part of the Old World Monkeys (hereafter referred to as OWmISG15) (see Table 1).

**Table 1:** Species classification of the various studied ISG15 orthologues with the relevant abbreviations.

Order	Family	Species	
Primates	Hominidae (HOm)	*Homo sapiens*	Human (Hu)
		*Pan troglodytes*	Chimpanzee (Cpz)
	Cercopithecidae or Old World Monkeys (OWm)	*Macaca mulatta*	Rhesus Monkey (Rhm)
		*Cercopithecus aethiops (also Chlorocibus aethiops)*	African Green Monkey (Agm)
Rodentia	Muridae	*Mus musculus*	Mouse (Mo)

### OWmISG15 readily ISGylates human proteins

**Figure 2:**
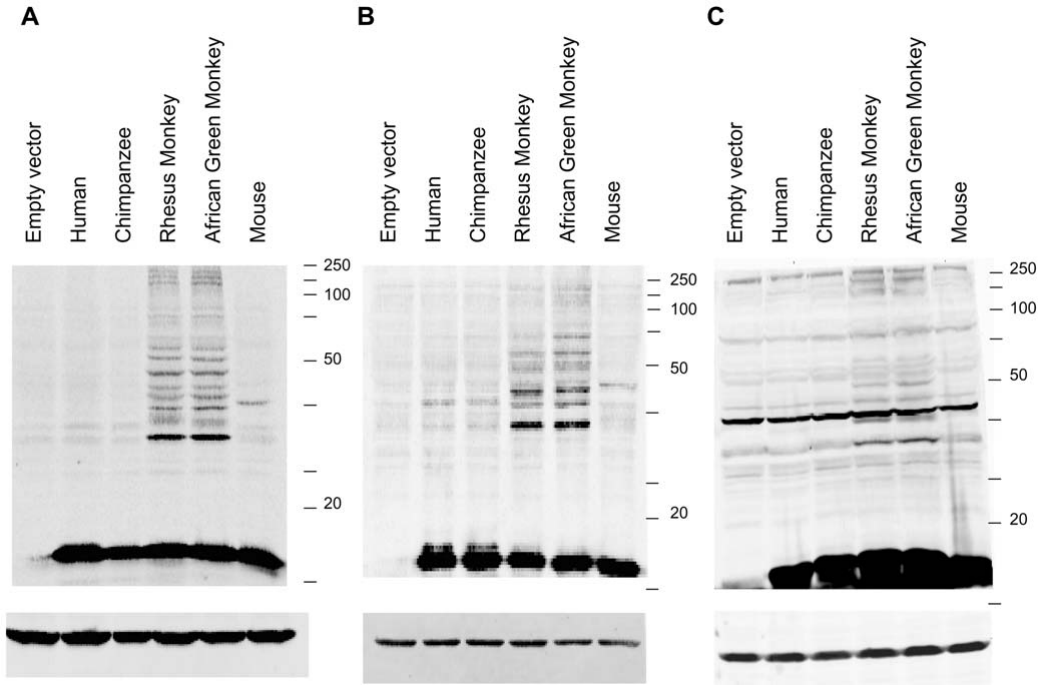
OWmISG15s conjugate more efficiently to target proteins. ISGylation pattern of FLAG-tagged Human, Chimpanzee, Rhesus Monkey, African Green Monkey and Mouse ISG15 in (A) human HekT cells, (B) monkey COS cells and (C) mouse N38 cells. Cells were only transfected with expression vectors for the indicated ISG15 orthologues, without extra stimulation. *Upper panels*: Western blot analysis on total cell lysates showing the total ISGylation pattern after ectopic expression of the indicated ISG15 orthologues as visualized by their FLAG-tag (anti-FLAG Ab). *Lower panels*: actin loading control (anti-actin Ab).

Total ISGylation patterns were visualized by Western blot analysis on total cell lysates. As can be seen in Figure 2a, AgmISG15 and RhmISG15 outperform HOmISG15 and MoISG15 in its ISGylation capacity in human Hek293T cells. MoISG15 also conjugates more favorably to human proteins compared to HuISG15, albeit not to the same extent as OWmISG15. To rule out the possibility that conjugation of ectopic HuISG15 could be hindered by competition with endogenous ISG15, the same blot was stripped and reprobed with an antibody recognizing Hu and OWmISG15, showing essentially the same results (data not shown). Similar findings were obtained in monkey COS cells and mouse N38 cells (resp. Figure 2b and Figure 2c). Note that all these experiments were performed without extra stimuli (e.g. IFN treatment) or co-transfection of any Ubiquitinating enzyme meaning that in all tested cell-lines, target ISGylation by AgmISG15 could be achieved by endogenously available Ubiquitinating enzymes.

### Identification of novel ISGylation substrates

We wanted to address whether the observed differences in ISGylation capacity was only quantitative or also qualitative. Therefore, Tandem Affinity Purification (TAP) experiments were performed to identify ISGylated substrates by Hu and AgmISG15. To this end, a tandem proteinA binding domain and FLAG-tag, separated by a Tobacco Etch Virus protease cleavage domain was fused N-terminally to Hu or AgmISG15. Human fibroblast 2fTGH cells were stably transfected with these constructs, and 2 cell clones expressing TAP-tagged HuISG15 or TAP-tagged AgmISG15 with comparable expression levels were selected (see Figure S1). To rule out experimental variation, this experiment was also performed in transiently transformed HekT cells with either TAP-tagged HuISG15 or AgmISG15. As shown in Table 2, the TAP experiments identified substantially more ISGylation substrates of AgmISG15 compared to HuISG15 in human cell-lines. Details of the identified peptides can be found in Table S1. In addition to an overlap with the already described ISG15 substrates [30]–[34], we here report the Ub-Conjugating enzymes UbcH10, UbcH16 are UbcH17 as novel ISGylation substrates of AgmISG15.

**Table 2:** ISGylated targets identified by TAP purification and LC-MS/MS identification of AgmISG15 outnumber those of HuISG15.

		Alternative name(s) Abbreviation	Isoforms?	HuISG15	AgmISG15
**Ub-Activating Enzyme**	UbE1	Uba		0	15
**Ub-Conjugating Enzymes**	UbcH6	E2E1	Q969T4, AQ96LR5	0	1
	UbcH10	E2C		0	8
	UbcH13	E2N, bendless-like E2	Q5JXB2	1	19
	UbcH16	E2-25, HIP2		0	3
	UbcH17	E2T, HSPC150		0	1
	Myosin light polypeptide 6B		P60660	0	1
	Actin, cytoplasmic 1		P60709,P62736 ,P63261 ,P63267 ,P68032 ,P68133 ,Q6S8J3		
	0	1			
	Phosphoglycerate mutase 2	PGAM2	P18669	0	1
	40S ribosomal protein S10	RS10		0	1
	Ubiquitin			2	2
**Heat Shock Proteins**	HSP70-1	HSP71	lots, but on different peptides	3	21
	HSP70-5	GRP78		2	1
	HSP70-1L	HS71L		0	1
	HSP90 alpha	HSP86	P08238 (276–284)	0	1
	Heat shock cognate 71 kDa protein	HSP7C		0	1

The numbers of MS/MS spectra identified with the different ISG15 orthologues are presented in the right columns.

**Figure 3:**
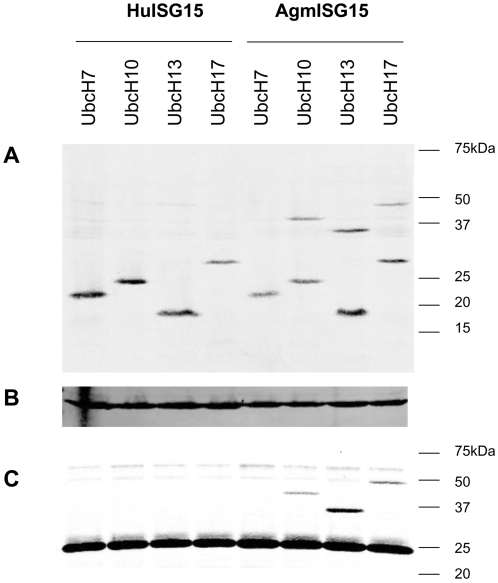
AgmISG15, but not HuISG15, efficiently ISGylates UbcH10, H13 and H17. V5-tagged UbcH7, H10, H13, H17 proteins were co-expressed in HekT cells with either FLAG-tagged Hu- or AgmISG15. (A) Total cell lysates were boiled in a SDS boiling buffer containing β-ME and loaded on a SDS-PAGE. The UbcH proteins were visualized by their V5-tag (Anti-V5 Ab). (B) Staining of blot (A) with anti-actin Ab as loading control. (C) Co-immunoprecipitation experiment with anti-FLAG affinity gel. The bound proteins were boiled in an SDS buffer containing reducing agents prior to separation by SDS PAGE. The immunoprecipitated UbcH proteins were visualized by an anti-V5 Ab.

The cDNAs of the Ub-Conjugating enzymes UbcH6, 7, 8, 10, 13, 16 and 17 were isolated by RT-PCR from 2fTGH cells and linked to a C-terminal V5-tag. These constructs were transfected in HekT cells together with either a mock construct, HuISG15 or AgmISG15. UbcH7 was included as a control, as it was neither in our experiments nor in others identified as a substrate for ISGylation. Western blot analysis on total cell lysates containing β-ME confirmed ISGylation of UbcH10, H13 and H17 with AgmISG15 but not with HuISG15, as seen by a 15 kDa shift upon staining with anti-V5 Ab detecting the ectopic expressed UbcH proteins (Figure 3a,b and Figure S2a). In addition, co-immunoprecipitation experiments were performed by binding ISGylated proteins through ISG15's FLAG-tag to an anti-FLAG affinity gel. Also here, the ISGylation of UbcH10, H13 and H17 by AgmISG15 could be established (Figure 3c). Worth mentioning, when the co-immunoprecipitated samples were obtained in a more concentrated way, a faint band of co-immunoprecipitated UbcH13 and UbcH17 could be seen with HuISG15 (Figure S2b), hinting at a qualitative rather than quantitative difference in ISGylation between Hu and AgmISG15.

Of note, all the experiments were performed without co-transfection of UbE1L and/or UbcH8 or IFN stimulus. Previous studies using Western blot analysis in HekT cells revealed Hu or MoISG15 conjugation to substrates such as UbcH13 only upon co-transfection of at least UbE1L -and generally also UbcH/M8- or upon IFN stimulation. Moreover, in most cases an additional immunoprecipitation or pull-down purification step was required [27], [28], [30]–[33]. Alternatively, experiments were performed in USP18-deficient Murine Embryonic Fibroblasts [30], [34].

### Mapping the determinants involved in efficient ISGylation

**Figure 4:**
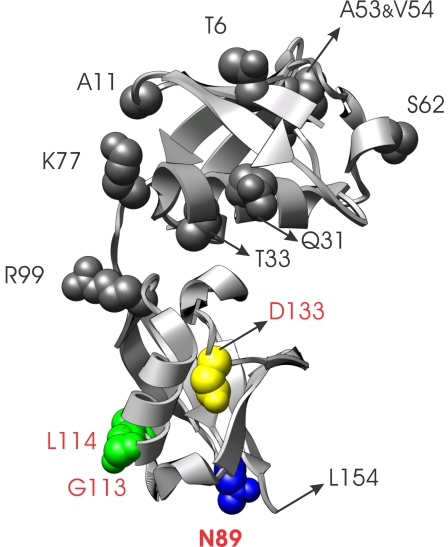
Crystal structure of HuISG15 with indication of the residues different in AgmISG15. Residues that differ between HuISG15 and AgmISG15 are shown as space filing spheres. Colored residues are dissimilar residues situated in or near the interaction site with UbE1L predicted by Narasimhan et al. [35].

We next replaced the differing amino acids in HuISG15 by their AgmISG15 counterparts (see Figure 1 and 4). Four of these residues (i.e. residues 89, 113, 114 and 133) are situated near the interaction interface between ISG15 and its Activating enzyme, UbE1L, predicted by Narasimhan et al. [35] (red highlighted in Figure 4). The N83S and S94N allelic variants of HuISG15 were also included. Both conjugation to various UbcHs and total ISGylation patterns of different HuISG15 mutants were tested.

**Figure 5:**
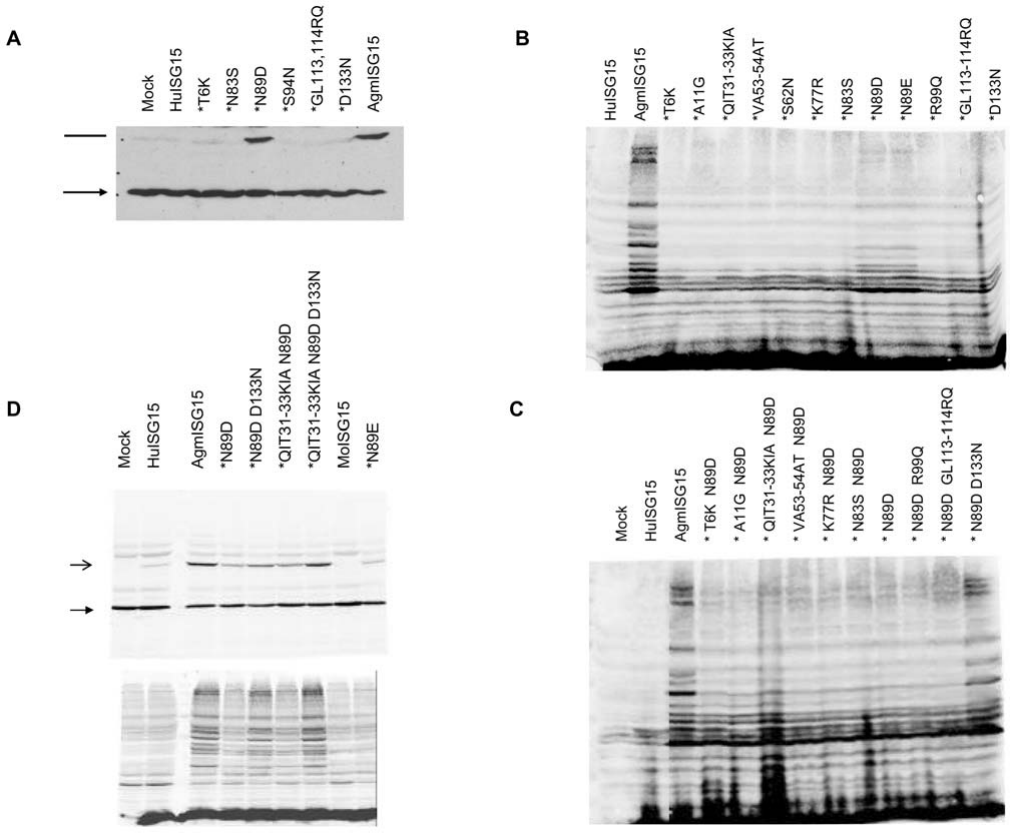
Mutation of residue 89 in HuISG15 to Asp greatly enhances its conjugation capacity, which could be further boosted by mutations of QIT31-33KIA and D133N. *(A* *)* HuISG15 N89D mutant significantly increases its ISGylation capacity to UbcH proteins. HekT cells were transiently transfected with a vector encoding V5-tagged UbcH10 together with either empty vector or a vector expressing HuISG15 or variants (indicated with asterisk) or AgmISG15. Total cell lysates were prepared in a buffer containing β-ME. UbcH10 protein is revealed by its V5-tag. Closed arrow indicates the unconjugated form of UbcH10, open arrow indicates the ISG15-conjugated form of UbcH10. *(B)* Only mutation of residue 89 in wild type HuISG15 to an acidic amino acid is able to enhance its ISGylation activity. HekT cells were transiently transfected with vectors encoding the indicated FLAG-tagged HuISG15 variants (indicated with asterisk) each holding one amino acid substitution, FLAG-tagged wt Hu- or AgmISG15. Total cell lysates were prepared in a buffer with reducing agentia. The conjugation competence of the different ISG15 proteins is exposed by their FLAG-tag. Equal loading was confirmed by Ponceau S staining (not shown). *(C)* Additional mutation of QIT31-33KIA or D133N further boosts ISGylation by HuISG15 N89D. Same as (B) but with HuISG15 N89D variants with an additional amino acid substitution. *(D)* The triple HuISG15 QIT31-33KIA N89D D133N variant is as efficient as AgmISG15 in UbcH protein ISGylation. *Upper panel*. HekT cells were transiently transfected with a plasmid encoding V5-tagged UbcH17 protein together with either an empty vector or a vector expressing FLAG-tagged Hu-, Agm-, MoISG15 or a HuISG15 variant (with asterisk). Western blot analysis on total cell lysates was performed under reducing conditions using anti-V5 Ab. Closed arrow indicates the unconjugated form of UbcH17, open arrow indicates the ISG15-conjugated form of UbcH17. *Lower panel* The blot was stripped and reprobed with anti-FLAG Ab showing the global ISGylation pattern. Equal loading was controlled by staining with Ponceau S staining (not shown).

The effect of mutating HuISG15 residues situated near the predicted UbE1L interface and the different allelic variants on conjugation to UbcH proteins is shown in Figure 5a and S3a. Strikingly, the single HuISG15 N89D variant displayed a greatly enhanced ISGylation efficiency. No effect was seen for all other mutants, including the N83S or S94N human allelic variants. Note that also in this experiment the cell lysates were boiled in SDS loading buffer containing β-ME. The slower migrating band differs 15 kDa from the unconjugated form of the UbcHs, and is thus the result of ISG15 bound by an isopeptide linkage, not by a thiolester bond. As with AgmISG15, no co-transfection of any Activating or Conjugating enzyme was required to visualize its conjugation by Western blot analysis on total cell lysates in HekT cells.

A more detailed study including all differing residues (and also the N89E variant as it occurs in mice), demonstrated that only mutation at position 89 affected the total ISGylation pattern. However, the effect of this mutation is only intermediary as compared with AgmISG15 (see Figure 5b). We next combined the HuISG15 N89D variant with additional mutations. As shown in Figure 5c, mutation of D133N and QIT31-33KIA in the HuISG15 N89D variant further enhanced its ISGylation in human HekT cells. Finally, the triple HuISG15 mutant N89D, D133N and QIT31-33KIA displayed an ISGylation efficiency comparable to the monkey orthologues (see Figure 5d and S3b).

### AgmISG15 can be activated by UbE1

**Figure 6:**
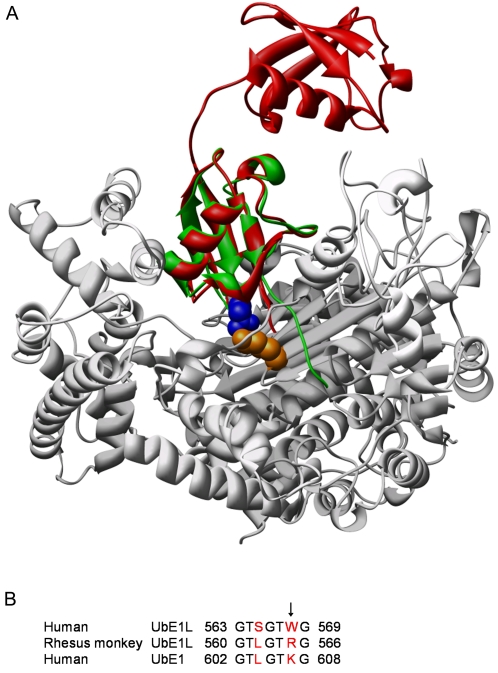
Residue 89 in ISG15 is situated in the predicted contact area with its Activating enzyme. *(A)* ISG15 (red ribbons) overlaid to Nedd8 (green ribbons) bound to its Activating enzyme AppBp1-Uba3 (grey ribbons). Residue 89 (dark blue) of ISG15, makes contact with residues in the two β-strands (shown in brown) of the Activating enzyme that precedes its C-terminal domain. K193 in Uba3 (orange) is located very close to N89, and corresponds to W568 in human UbE1L. The other residues of ISG15 with an important effect on total ISGylation, situated at positions 133 (yellow) and 31–33 (cyan blue), do not make contact with UbE1L and are likely to be involved in another feature of the ISGylation process. *(B)* HuUbE1L was aligned with HuUbE1 and RhmUbE1L (Ensembl peptide sequence ENSMMUP00000027609). The region 563–569 in HuUbE1L shows two substitutions (S565L and W568R, red coloured) in the corresponding region of RhmUbE1L, which better resembles the corresponding region in HuUbE1 (K607).

The interaction interfaces of Nedd8 bound to its Activating Enzyme, AppBp1-Uba3, [36] and SUMO bound to Sae1-Sae2 [37] have recently been mapped and show a remarkable high degree of similarity. We built a similar homology model for the UbE1L-ISG15 complex (Figure 6a), based on the crystal structure of the AppBp1-Uba3-Nedd8-ATP complex [38]. This superposition brings the critical residue 89 in ISG15 in the interaction interface with UbE1L. Therefore, we compared the sequences of Hu and OWmUbE1L in their predicted interfaces with ISG15 and found an interaction region displaying significant substitutions (i.e. the sequence 563-GTSGTWG-569 in HuUbE1L corresponding to 560-GTLGTRG-566 in OWmUbE1L). Residue W568 in HuUbE1L juxtaposes to ISG15 N89 (Figure 6a). This residue is substituted with an Arg residue in OWmUbE1L. Strikingly, the corresponding sequence in OWmUbE1L shows high similarity with HuUbE1 (Figure 6b).

**Figure 7:**
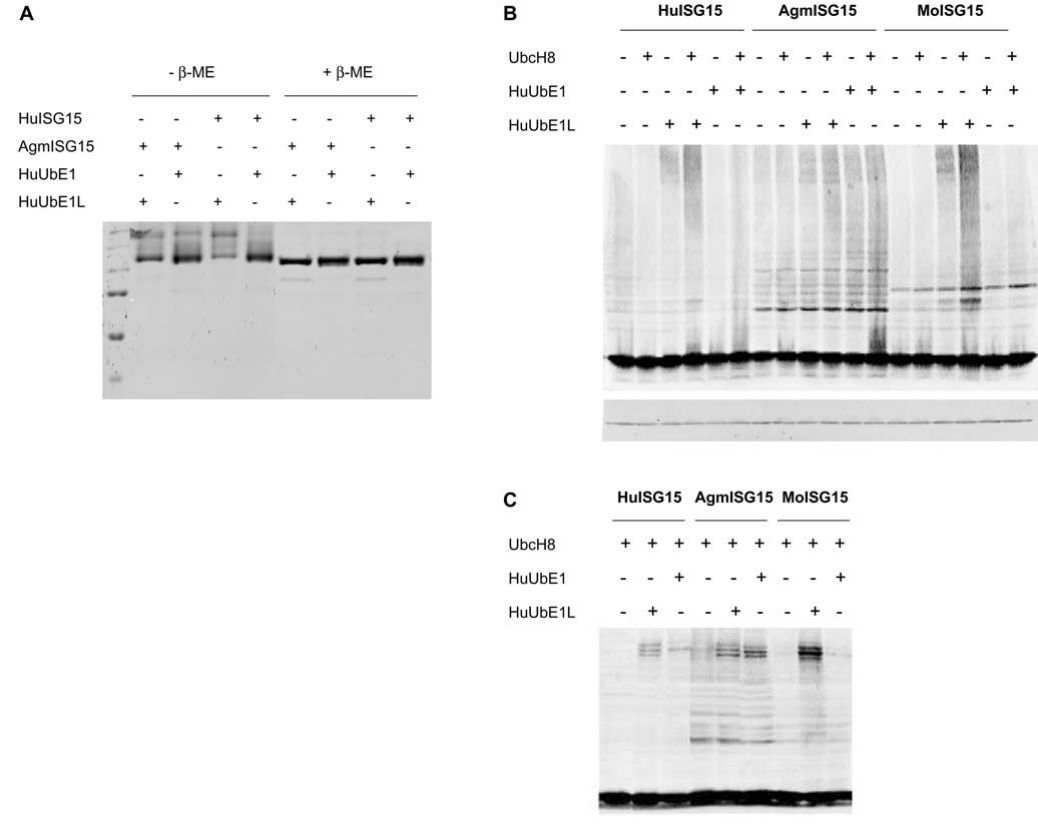
Promiscuity of the Activating enzyme of Ub in activating AgmISG15. (A) *In vitro* assay revealing the capability of AgmISG15 to form thiolester bonds with UbE1, contrary to HuISG15. 2.5 µM UbcH8 was incubated with 200 nM of either UbE1 or UbE1L, and 7.5 µM of either mature Hu- or AgmISG15 under conditions as described in Materials and Methods. The samples were divided in two aliquots; and were subjected to SDS-PAGE under non-reducing or reducing conditions. The proteins were stained with IRDye Blue protein stain and visualized using the Odyssey infrared imaging system. The extra band representing thiolester formation between UbE1 and AgmISG15 is flanked by two asterisks. These thiolester bonds are disrupted by the addition of reducing agent (here β-ME). (B) UbE1 can efficiently activate AgmISG15, but not Hu or MoISG15. HekT cells were transfected with an empty vector, or combinations of vectors encoding HuUbE1L, HuUbE1 and UbcH8 together with HuISG15, AgmISG15 or MoISG15 as indicated. The total ISGylation patterns were evaluated by Western blot analysis using the FLAG tag of the ISG15 proteins. Equal loading was confirmed by actin staining. (C) Same as (B), but the experiment was performed in COS cells. Only co-transfections with UbcH8 are shown.

This observation prompted us to test whether AgmISG15 could be activated by HuUbE1. We performed an *in vitro* assay analyzing the loading of either HuISG15 or AgmISG15 with HuUbE1 or HuUbE1L. Thiolester formation of both ISG15 orthologues with HuUbE1L was observed, as expected. Importantly, as anticipated from the sequence similarity, HuUbE1 was also found to be able to form thiolester bonds with AgmISG15, whilst no loading of HuISG15 could be seen (Figure 7a). The thiolester nature of the bond was verified by addition of β-ME. Western blot analysis evaluating the total ISGylation pattern further confirmed this alternative activation pathway for AgmISG15 (Figure 7b for the experiment in HekT cells, Figure 7c in COS cells). The cells were transfected with combinations of an empty vector or vectors encoding HuUbE1L, HuUbE1 and UbcH8 together with FLAG-tagged HuISG15, AgmISG15 or MoISG15. Visualising the ISGylation pattern on the different samples only observed significantly enhanced ISGylation by Hu and MoISG15 upon ectopic expression of UbE1L. A beneficial effect on Hu/MoISGylation efficiency upon ectopic expression of UbE1 was minimal. By contrast, even with the higher basal ISGylation level of AgmISG15, overexpression of both UbE1L and UbE1 were able to further markedly enhance its conjugation capacity.

## Discussion

The *isg15* gene emerged upon the duplication of an Ub dimer and insertion in an IFN-controlled area [39]. In sharp contrast with Ub itself, of which 72 of the 76 amino acid are invariant between animals, plants and fungi [40], ISG15 is not well conserved between species [2].

This variability in ISG15 sequence may indicate a redundant function of ISG15. Alternatively, it could point towards a role for ISG15 in the immune system, since high interspecies sequence divergence is very often associated with genes involved in host defense. Organism-specific niche occupation may lead to distinct repertories of invading pathogens causing species-specific pressures for adaptation of the host immune system [41], [42]. This increased evolutionary rate in immune genes may be further enhanced by the high mutation rates of the pathogens [43]. In line with the assignment of a specific role for ISG15 in host defense, naturally occurring loss-of-function ISG15 mutants have not yet been observed sofar, whereas a duplication of the ISG15 gene has been seen in crucian carp [44]. Although the precise function of ISG15 remains enigmatic, several lines of evidence point to its role as an antiviral agent. ISG15 is one of the most prominently induced genes upon type I IFN treatment, and several viruses such as Influenza B and SARS have developed mechanisms to counteract ISG15 function [9], [20]. ISG15 was also found to be critical for the IFN-mediated inhibition of HIV replication and release [25]. Moreover, although ISG15 knock-out mice were initially found to be as vulnerable to viral infections as their wild type countermates [45], a subsequent study discovered ISG15 knock-out mice to be more vulnerable to Influenza, Herpes and Sindbis viral infection [24].

A key finding of this report is the significant difference in ISGylation capacity between ISG15 orthologues. In human cells, protein modification by ISG15 of OWm greatly outperforms that of Hu and MoISG15. Mutation of the residues in HuISG15 to the corresponding residues in the OWm orthologue mapped amino acid 89 as a critical residue for ISGylation. The occurrence of an acidic residue (preferably an Asp in human cells and Glu in mouse cells) at this position greatly enhances its ISGylation capacity. The Asp residue at position 89 in OWmISG15 also occurs in sheep and cows. In rodents and fish, this residue is a Glu residue. The incidence of the unfavorable Asn residue at position 89 for ISG15 modification in HuISG15 is also found in chimpanzee and dog ISG15. However, notwithstanding the greatly enhanced ISGylation capacity by mutation of the single Asn 89 residue in HuISG15 to an Asp residue, the overall ISGylation pattern in human HekT cells was still intermediary compared to AgmISG15. Additional mutations were created in the HuISG15 N89D mutant. Mutation of D133N and QIT31-33KIA further increased its ISGylation capacity. The triple HuISG15 mutant N89D D133N QIT31-33KIA proved to be as effective as AgmISG15 in conjugating target proteins in HekT cells. The use of this hyperefficient ISG15 variant may help to unravel the physiological function of ISG15.

The Ub(L) modification process is initiated by its dedicated Activating enzyme, a crucial step which is also described to dictate selectivity. Ub-Activating enzymes are composed of three modular domains: an N-terminal domain which targets ATP and Ub(L)-binding (residues 1–595 in UbE1L), a second domain harboring the catalytic Cys residue (embedded in residues 596–880) and an C-terminal UbL-fold domain (residues 914–1013), which selects for and binds to the cognate Ub-Conjugating enzymes [8], [46].

Based on interaction interfaces of Nedd8 bound to AppBp1-Uba3 [36], Narasimhan and colleagues predicted 7 hot spot residues on ISG15 constituting the interface with UbE1L. Three of these residues show a high degree of conservation among the different Ub(L)s (i.e. R92, E132 & R153 in ISG15), 4 other residues are presumed to confine specificity for UbE1L (R87, K90, W123 & F149) [35]. We built a similar homology model for the UbE1L, using the crystal structures of the AppBp1-Uba3-Nedd8-ATP complex. Structure superposition shows that residue 89 corresponds to L8 in Nedd8 (Figure 6a), a well-conserved residue that is also found in Ub (Figure 1). Mutation of L8 in Ub significantly reduced its target conjugation ability [47], and in yeast this residue proved to be essential for viability [48]. This suggests that residue 89 in ISG15 and L8 in Ub have a similar important role in conjugate formation. L8 in Nedd8 is part of a hydrophobic patch (L8, I44, V70) that interacts with V323 and Y331 of Uba3, embedded in the two β-sheets preceding its C-terminal domain. Mutation of these residues greatly reduced Nedd8 adenylation and consequently conjugation [36]. However, residues L8, I44 and V70 in Nedd8 correspond with the non-hydrophobic residues N89, T125 and N151 in ISG15, suggesting different types of interaction in this region. The two Uba3 β-strands correspond to the region 880–895 in the UbE1L model, but the alignment in this region is too poor to allow reliable modeling of the actual interactions. Moreover, residues 152-LRL-154, which are part of the C-terminal region of ISG15 juxtapose to N89 (Figure 4). This region aligns with 71-LRL-73 in Ub which was described to be of high importance for UbE1 binding affinity and for substrate specificity [49]. Our model suggests that residue 89 makes a contact with R565 in OWmUbE1L, which lies in the N-terminal domain, known to select for a specific UbL [36], [37]. In HuUbE1L, this Arg is replaced by a Trp. Strikingly, the sequence 560-GTLGTRG-566 in OWmUbE1L is very similar to the corresponding region 602-GTLGTKG-608 in HuUbE1 (see also Figure 6b). Based upon this observation, we experimentally demonstrated that HuUbE1 could efficiently activate AgmISG15, in contrast to Hu and MoISG15. This non-redundant role for UbE1L in activating MoISG15 is also in line with the loss of MoISG15 conjugation in UbE1L knock-out mice [50]. Unlike UbE1L, which is IFN-inducible and has a tissue and cell-line specific expression profile [51], UbE1 is ubiquitously expressed. Taken together, our findings support a role for residue 89 in ISG15 as a hot spot residue in the interface with its Activating enzyme, thus providing an explanation for the hyperISGylation seen for OWmISG15. Recently, with the discovery of UbE1L2 (Uba6) as the Activating enzyme for FAT10 and as a second Activating enzyme for Ub [7], [8], the theory of an Activating enzyme harboring unilateral assignment for a specific Ub(L) was overthrown. Here, we describe promiscuity of UbE1 for OWmISG15 activation. This is the first report of ISG15 being activated by another Activating enzyme than UbE1L. Moreover, to the best of our knowledge, this is also the first report of an UbL -other than Ubiquitin- being activated by UbE1.

In contrast, homology modeling and structural superpositions do not predict a direct role for residue D133 or residues QIT31-33 of ISG15 in the activation step (Figure 4 and 6a). The D133 residue is part of a ridge of negatively charged residues along the ISG15 molecule [35]. The function of this ridge is unclear. D133 in HuISG15 corresponds to a negatively charged residue in Ub, SUMO, Nedd8, RUB1, Apg12 (all Asp residues) and Urm1 (occupied by a Glu residue). Models based on the AppBp1-Uba3-Nedd8-ATP complex or the Sae1-Sae2-SUMO-Mg-ATP complex, do not predict direct contact of residue 133 in ISG15 with Ube1L. The same hold true for residues 31–33. This suggests that mutations at position 31–33 and 133 influence total ISGylation by a different mechanism than mutation at position 89. This must occur later in the ISGylation process, since no effect of these mutations can be observed in the absence of the N89D mutation. Of note, notwithstanding their position on different Ub domains in ISG15, residues 31, 33 and 133 are situated at the same face of the molecule (see Figure 4) close to the electronegative ridge. As the total protein ISGylation results from the balance between conjugation and de-conjugation, mutation of QIT31-33 to KIA and D133 to N in HuISG15 N89D conceivably could affect recognition or activity by (a) discriminate DUB(s), retaining the targeted protein in the ISGylated state. In line with this hypothesis, a role has been described for the N-terminal Ub fold of ISG15 in the efficiency of DUB recognition/activity of the SARS coronavirus papain-like protease [52].

In this study we also present the Ub-Conjugating enzymes UbcH10, H16 and H17 as new ISGylation targets. UbcH10 (UbE2C) acts as an UbcH for the Anaphase Promoting Complex (APC), promoting cyclinA degradation and mitotic exit. During the G1 phase, UbcH10 is auto-ubiquitinated allowing re-accumulation of cyclinA and entry in the S phase [53]. UbcH10 is often referred to as the cancer-related UbcH as it is markedly overexpressed in the majority of cancerous cell lines [54]. Recently, also UbcH16 (E2-25K or HIP2) has been identified as an APC-dependent UbcH promoting Ub K48-linked chain extensions on pre-attached Ubs [55]. UbcH17 (UbE2T) has recently been identified as the UbcH essential to ubiquinate FANCD2, which needs to be ubiquitinated in order to bind BRCA2 and take part in the DNA repair process. UbcH17-depleted cells have abnormal chromosomes, characteristic for Fanconi anemia [56], [57]. These findings may help explain the role for ISG15 in anti-tumor defense.

In conclusion, we report that OWmISG15 more efficiently ISGylates proteins compared to human ISG15. It remains to be determined whether this reflects merely quantitative or also qualitative differences in the role of ISG15 among species. As ISG15 is implicated in antiviral protection against various viruses including SARS, Influenza, Hepatitis C and HIV, these differences in ISG15 conjugation efficiency may help explain human sensitivity to various viruses.

## Materials and Methods

### Constructs

Human ISG15 (allelic variant with N at position 83 and S at position 94) was isolated from cDNA of Hek293T cells, African Green Monkey ISG15 from cDNA of Vero cells and Mouse ISG15 from cDNA of L929SA cells.

All HuISG15 mutants as well as the other human allelic variants (N83S and S94N) were created through site-directed mutagenesis (Stratagene). Chimpanzee ISG15 was obtained by site-directed mutagenesis of HuISG15 (S21N mutation), Rhesus Monkey ISG15 was obtained by 2 consecutive site-directed mutations on AgmISG15 (S21N and K77R mutation).

### Cell culture and transfection protocol

All cell-lines were cultured in a 8% CO2 humidified atmosphere at 37°C and grown in Dulbecco's modified Eagle's medium (Invitrogen) with 10% fetal calf serum (Cambrex Corp.).

For standard ISGylation experiments in Hek239T cells, 3,5×10^5^ cells were seeded the day before transfection in 6-well plates and transfected for 6 h using a standard calcium phosphate precipitation procedure. Typically 0.75 µg of ISG15variant, if indicated supplemented with 0.25 µg E1 and 0.5 µg E2 or, applicable supplemented with 0.75 µg substrate was used in a total volume of 250 µL DNA/CaPO4-transfection mixture. FugeneHD (Roche) was used for transfection in COS cells and Lipofectamine2000 (Invitrogen) for transfection in N38 cells. Since FugeneHD induces a minimal IFN response, this reagent was exceptionally used for the Hek293T transfections in Figure 7b. However, no differences could be seen between this transfection reagent and the calcium phosphate precipitation method regarding the ISGylation difference between Hu and AgmISG15.

TAP purification experiments were performed in 2fTGH cells stably expressing either Hu or AgmISG15 and HekT cells transiently expressing either Hu or AgmISG15 with an N-terminal TAP-tag. Single colonies of the 2fTGH cells were selected upon co-transfection of the ISG15 constructs with a puromycin resistance marker and selection on 3 µg/mL puromycin. In order to check the expression levels of the TAP-ISG15 proteins compared to the endogenous ISG15 levels, the different stable cell-lines were stimulated for 26 h with IFNβ (Peprotech) prior to Western blot analysis.

### Lysate preparation and Western blot analysis

Two days after transfection, cells were lysed with 150 µL 2× SDS loading buffer (62 mM Tris HCl pH 6.8, 2% SDS, 8% glycerol, 5% β-ME, 0.01% brome phenol blue) and homogenized on a Qiashredder mini spin column (Qiagen). 35 µL of the boiled lysate was loaded on a SDS-polyacrylamide gel. Blotting efficiency was checked using Ponceau S staining (Sigma). The precision plus protein standard all blue (Biorad) was used as molecular weight marker. FLAG-tagged proteins were revealed using a 1/8000 dilution of anti-FLAG M2 mouse monoclonal Ab (Sigma), V5-tagged proteins by 1/5000 dilution of anti-V5 mouse monoclonal Ab (Invitrogen). Hu and OWmISG15 could be detected by an anti-human ISG15 rabbit polyclonal Ab (Abcam). Human ISG15 was also detected with the anti-human ISG15 mouse monoclonal Ab, a generous gift from Dr. E. Borden. Anti-human actin rabbit polyclonal Ab (Sigma) 1/3000 diluted was applied as control loading. Either goat anti-mouse IRDye® 800CW or anti-rabbit IRDye® 680 (LI-COR® Biosciences) was used as secondary Ab. Targeted proteins on the blots were visualized using the Odyssey® infrared imaging system (LI-COR® Biosciences).

### TAP purification and mass spectrometry

The 2fTGH or Hek293T cells expressing either TAP-tagged AgmISG15 or HuISG15 were lysed in cell lysis buffer (50 mM Tris-HCl pH 8, 10% glycerol, 1% NP40, 150 mM NaCl, 5 mM NaF, 5 µM ZnCl_2_, 1 mM Na_3_VO_4_, 10 mM EGTA, Complete™ Protease Inhibitor Cocktail (Roche)). The insoluble fraction was spun down and the supernatant was incubated with IgG sepharose (Amersham Biosciences) overnight. The beads were washed three times with washing buffer (2 mM Tris-HCl pH 7.5, 5% glycerol, 0.1% NP40, 150 mM NaCl) and twice with TEV (Tobacco Etch Virus) protease cleavage buffer 1 (10 mM Tris-HCl pH 8, 150 mM NaCl, 0.1% NP40, 0.5 mM EDTA) and were then incubated with TEV protease in TEV protease cleavage buffer 1 for 2 hours. The beads were then spun down and the supernatant was incubated with anti-FLAG agarose (Sigma) in TEV protease cleavage buffer 2 (10 mM Tris-HCl pH 8, 150 mM NaCl, 0.1% NP40) for 2 to 4 hours. The anti-FLAG agarose beads were washed three times with washing buffer and incubated with 250 µg/mL FLAG peptide in FLAG elution buffer (2 mM Tris-HCl pH 7.5, 5% glycerol, 150 mM NaCl) for 10 min to elute for the FLAG-tagged proteins. The resulting peptide mixture was precipitated by addition of TCA to a final concentration of 10%. It was incubated overnight, centrifuged and washed with ice-cold acetone containing 0.05N HCl and dried. Pellets were re-dissolved in 3 µL 50 mM NH_4_HCO_3_ (pH 7.9) containing 8M ureum for 20min periodic vortexing, 21 µL 50 mM NH_4_HCO_3_ (pH 7.9) was added in aliquots to lower the final concentration of ureum to 1 M. The resulting peptide mix was digested in solution by trypsin and applied for nano-LC-MS/MS analysis as described before using a Waters Q-TOF mass spectrometer [58] or a Bruker Esquire HCT ion trap mass spectrometer [59].

The generated peptide fragmentation spectra were searched using the MASCOT database search engine (http://www.matrixscience.com) in the SwissProt database (taxonomy was set to human). The following MASCOT parameters were set. The enzyme setting was trypsin with a maximum of 1 missed cleavage allowed. Variable amino acid modifications that were allowed are acetylation (N-term), carbamylation (Lys and N-term), deamidation (Asn and Gln), formation of pyroglutamate (N-terminal Gln), oxidation of Met to its sulfoxides and propionamide modification of Cys. Allowed peptide and fragment ion mass tolerance were 0.3 Da (Q-TOF) or 0.5 Da (ion trap). MASCOT's instrument setting was “ESI-QUAD-TOF” (Q-TOF) or “ESI-TRAP” (ion trap) for calculating theoretical peptide fragmentation spectra.

Following database searching, only spectra that exceeded the corresponding MASCOT's threshold score for identify (set at the 95% confidence level) are here reported. The matching estimated false positive discovery rate is well acceptable and is only between 2 to 4% at the individual spectrum level as previously assessed [60].

### Co-immunoprecipitation procedure

2×10^6^ Hek293T cells were transfected with the indicated expression vectors. Cleared lysates (modified RIPA lysis buffer: 200 mM NaCl, 50 mM Tris-HCl pH 8, 0,05% SDS, 2 mM EDTA, 1% NP40, 0,5% DOC, Complete™ Protease Inhibitor Cocktail (Roche)) were prepared two days after transfection. The samples were incubated with anti-FLAG M2 affinity gel (Sigma). After immunoprecipitation, SDS-PAGE and Western Blotting, interactions were detected using an anti-V5 antibody (Invitrogen)

### Production and purification of Hu and AgmISG15 proteins

Mature ISG15 (with removed C-terminal peptide) was cloned in the pTYB1 vector and purified with the IMPACT™ procedure (Intein Mediated Purification with an Affinity Chitin-binding Tag) (New England Biolabs) When the cultures reached an OD600 of 0.6, isopropyl β-d-thiogalactopyranoside (IPTG) was added to a final concentration of 0.3 mM, and were grown at 15°C overnight. The next day, cells were removed from the medium by centrifugation. The cell pellets were resuspended in ice-cold cell Lysis Buffer (20 mMTris pH 7.5, 500 mM NaCl) and disrupted mechanically by sonication. After removal of cell debris by centrifugation, the clarified lysate was loaded onto a chitin column (equilibrated with 10 volumes of the column buffer (20 mM Tris, 500 mM NaCl, 1 mM EDTA, pH 9.0). Three bed volumes of the cleavage buffer (20 mM Tris, 1 mM EDTA, 50 mM DTT, pH 9.0) were loaded onto the column, and incubated overnight. The ISG15 protein was eluted from the column using twice 4ml column buffer and dialysed against 3 times 5 L PBS overnight.

The concentration of the protein was determined by the Bradford assay. 30 µL was loaded on a 12% SDS page gel and silver stained to check the quality and purity of the ISG15 proteins. Purified recombinant UbE1, UbE1L, UbcH8, UbcH10 and UbcH13 were purchased (Boston Biochem).

### In vitro assays ISG15 conjugation assay

The *in vitro* thiolester reactions were performed based on the ISG15 conjugation initiation kit (Boston Biochem). All samples were prepared in 20 µL containing 50 mM HEPES pH 8.0 and 100 mM NaCl. The concentration of the recombinant proteins is indicated in the Figure Legend. Reaction was started by the addition of 1 mM Mg-ATP (Boston Biochem), followed by a 45 min incubation at 37°C. The reaction was stopped by the addition of 20 µL of 2× SDS loading buffer (62 mM Tris HCl pH 6.8, 2% SDS, 8% glycerol, 0.01% brome phenol blue), with or without the addition of 5% β-ME (710 mM) as indicated in the Figure and/or Figure Legend. Proteins were separated by SDS-PAGE and were stained with either IRDye Blue Protein Stain (Licor) or by silver staining.

## Supporting Information

Figure S1 The selected stable 2fTGH cell clones express comparable levels of the TAP-tagged ISG15 orthologues. 2fTGH cells were stably transfected with the indicated TAP-tagged constructs as described in Materials and Methods. Four clones were selected for the TAP experiments, 2 clones expressing TAP-tagged HuISG15 and 2 clones expressing TAP-tagged AgmISG15. One cell-line stably expressing TAP-tagged PRMT (Protein Arginine N-Methyltransferase) was used as a control (lane 2). The different cell-lines were seeded at the same density in 12-well plates. 16 h after seeding, the cell-lines were stimulated with IFNβ(1 ng/ml). One non-stimulated (ns) control was included (lane 1). 26 h after IFNβtreatment, cell lysates were prepared and separated by SDS PAGE. (A) Western blot using anti-FLAG Ab, revealing the TAP-tagged constructs. Open arrow indicates the ectopic expressed TAP-tagged ISG15 constructs. The PRMT control construct is a bigger protein. (B) Western blot using anti-HuISG15 Ab (gift of Dr. E Borden). Open arrow indicates the ectopic expressed TAP-tagged ISG15 constructs (note the weaker cross-species recognition of AgmISG15 by the antibody). Closed arrow indicates the induced endogenous ISG15 as a result of the IFN stimulation.
(0.25 MB TIF)

Figure S2 AgmISG15, but not HuISG15, efficiently ISGylates UbcH10, H13 and H17. (A) Plasmid vectors encoding V5-tagged UbcH7, H8, H10, H13, H17 proteins were transfected in HekT cells together with either a mock construct, HuISG15 or AgmISG15. Total cell lysates were boiled in a SDS boiling buffer containing β-ME and loaded on a SDS-PAGE. The UbcH proteins were visualized by their V5-tag. The arrow indicates the 15 kDa difference in molecular mass of the ISGylated form of the UbcH protein. (B) Same co-immunoprecipitation experiment as in Figure 3b, but samples were more concentrated. A faint band of co-immunoprecipitated UbcH13 and UbcH17 with HuISG15 is here observed.
(0.64 MB TIF)

Figure S3 Mutation of residue 89 in HuISG15 to an Asp greatly enhances UbcH6, H10, H13 and H17 ISGylation. The triple HuISG15 mutant shows an ISGylation pattern comparable to AgmISG15. (A)HekT cells were transiently transfected with the plasmids encoding the indicated V5-tagged UbcH proteins together with either empty vector or FLAG-tagged HuISG15 or variants (indicated with asterisk) or AgmISG15. Total cell lysates were prepared in a buffer with reducing agentia. UbcH proteins were revealed by their V5-tag. The shown bands represent the ISGylated forms of the UbcH proteins. (B) Left panel. HekT cells were transiently transfected with plasmids encoding the indicated V5-tagged UbcH proteins together with either empty vector or FLAG-tagged Hu, Agm or Mo ISG15 or a HuISG15 variant (with asterisk). Revelation was with anti-V5 Ab. Closed arrows indicate the unconjugated form of the UbcH proteins. Open arrows show the position of the UbcH proteins conjugated by an isopeptide bond to the specified ISG15. Right panel The same blot was stripped and reprobed with an anti-FLAG Ab showing the global ISGylation pattern upon expression of the indicated ISG15 protein. Equal loading was confirmed by Ponceau S staining (not shown).
(2.03 MB TIF)

Table S1 Detailed description of the peptides identified as targets of Hu- or AgmISGylation upon TAP purification and LC-MS/MS identification.
(0.04 MB XLS)
