# Supplementary figures and images for: HyperISGylation of Old World Monkey ISG15 in Human Cells
Source: PLoS One. 2008 Jun 18;3(6):e2427. doi: 10.1371/journal.pone.0002427 (PMC2423471; doi:10.1371/journal.pone.0002427)

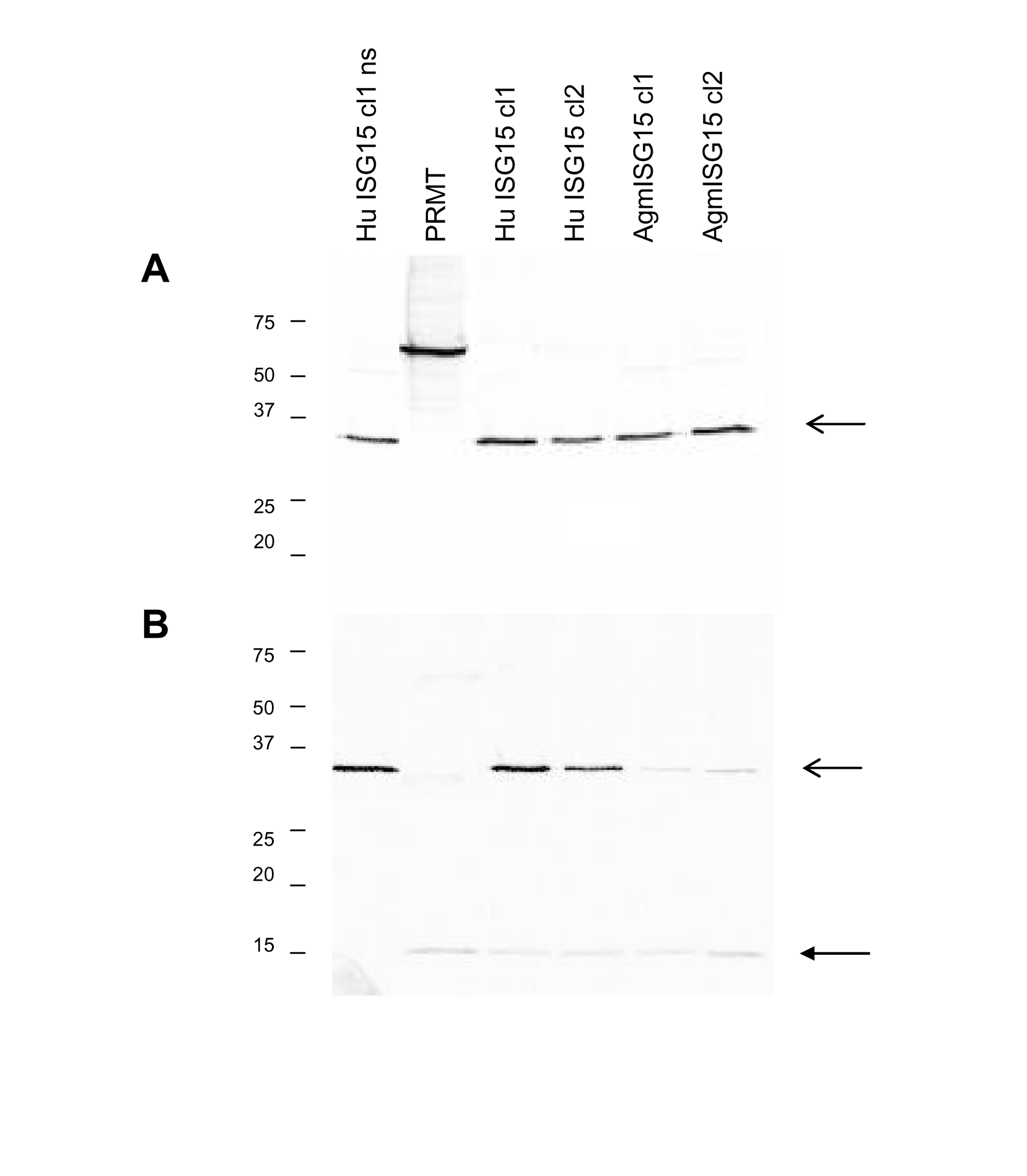

Supplement: Figure S1 — The selected stable 2fTGH cell clones express comparable levels of the TAP-tagged ISG15 orthologues. 2fTGH cells were stably transfected with the indicated TAP-tagged constructs as described in Materials and Methods. Four clones were selected for the TAP experiments, 2 clones expressing TAP-tagged HuISG15 and 2 clones expressing TAP-tagged AgmISG15. One cell-line stably expressing TAP-tagged PRMT (Protein Arginine N-Methyltransferase) was used as a control (lane 2). The different cell-lines were seeded at the same density in 12-well plates. 16 h after seeding, the cell-lines were stimulated with IFNβ(1 ng/ml). One non-stimulated (ns) control was included (lane 1). 26 h after IFNβtreatment, cell lysates were prepared and separated by SDS PAGE. (A) Western blot using anti-FLAG Ab, revealing the TAP-tagged constructs. Open arrow indicates the ectopic expressed TAP-tagged ISG15 constructs. The PRMT control construct is a bigger protein. (B) Western blot using anti-HuISG15 Ab (gift of Dr. E Borden). Open arrow indicates the ectopic expressed TAP-tagged ISG15 constructs (note the weaker cross-species recognition of AgmISG15 by the antibody). Closed arrow indicates the induced endogenous ISG15 as a result of the IFN stimulation. (0.25 MB TIF) [file pone.0002427.s001.tif]

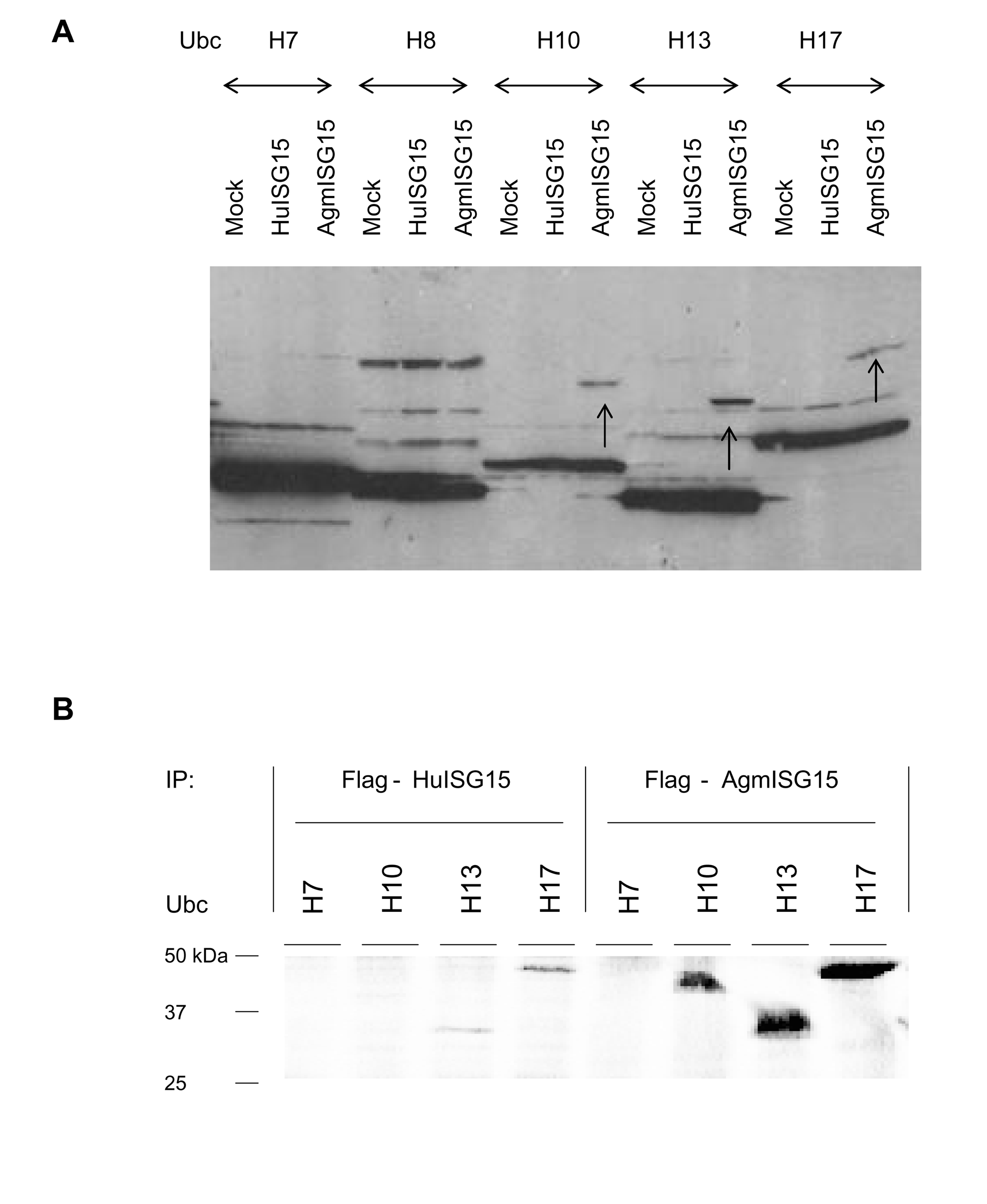

Supplement: Figure S2 — AgmISG15, but not HuISG15, efficiently ISGylates UbcH10, H13 and H17. (A) Plasmid vectors encoding V5-tagged UbcH7, H8, H10, H13, H17 proteins were transfected in HekT cells together with either a mock construct, HuISG15 or AgmISG15. Total cell lysates were boiled in a SDS boiling buffer containing β-ME and loaded on a SDS-PAGE. The UbcH proteins were visualized by their V5-tag. The arrow indicates the 15 kDa difference in molecular mass of the ISGylated form of the UbcH protein. (B) Same co-immunoprecipitation experiment as in Figure 3b, but samples were more concentrated. A faint band of co-immunoprecipitated UbcH13 and UbcH17 with HuISG15 is here observed. (0.64 MB TIF) [file pone.0002427.s002.tif]

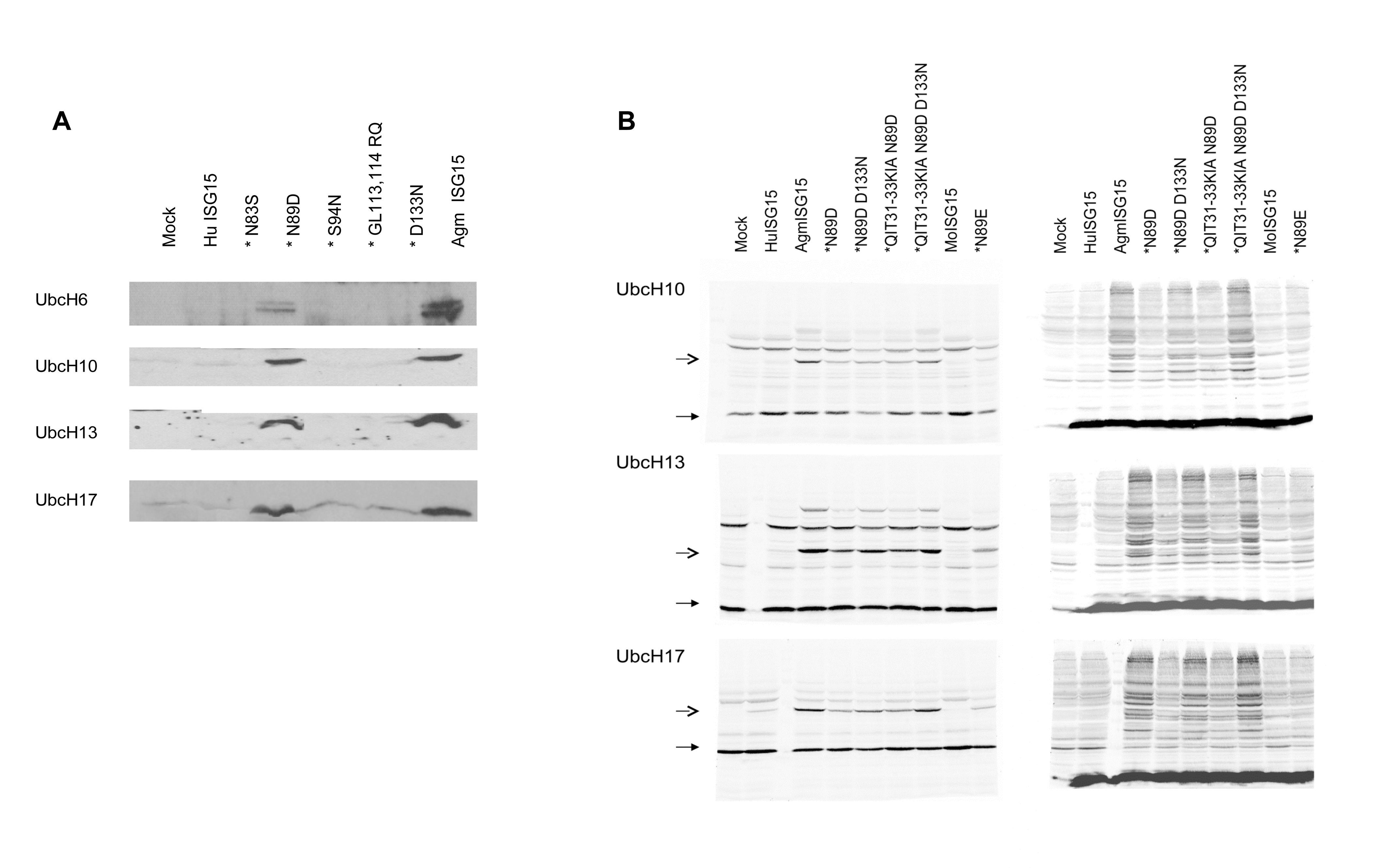

Supplement: Figure S3 — Mutation of residue 89 in HuISG15 to an Asp greatly enhances UbcH6, H10, H13 and H17 ISGylation. The triple HuISG15 mutant shows an ISGylation pattern comparable to AgmISG15. (A)HekT cells were transiently transfected with the plasmids encoding the indicated V5-tagged UbcH proteins together with either empty vector or FLAG-tagged HuISG15 or variants (indicated with asterisk) or AgmISG15. Total cell lysates were prepared in a buffer with reducing agentia. UbcH proteins were revealed by their V5-tag. The shown bands represent the ISGylated forms of the UbcH proteins. (B) Left panel. HekT cells were transiently transfected with plasmids encoding the indicated V5-tagged UbcH proteins together with either empty vector or FLAG-tagged Hu, Agm or Mo ISG15 or a HuISG15 variant (with asterisk). Revelation was with anti-V5 Ab. Closed arrows indicate the unconjugated form of the UbcH proteins. Open arrows show the position of the UbcH proteins conjugated by an isopeptide bond to the specified ISG15. Right panel The same blot was stripped and reprobed with an anti-FLAG Ab showing the global ISGylation pattern upon expression of the indicated ISG15 protein. Equal loading was confirmed by Ponceau S staining (not shown). (2.03 MB TIF) [file pone.0002427.s003.tif]
